# Benzo (A) pyrene exposure alters alveolar epithelial and macrophage cells diversity and induces antioxidant responses in lungs

**DOI:** 10.1016/j.toxrep.2024.101777

**Published:** 2024-10-18

**Authors:** Pooja Chauhan, Nitin Bhardwaj, Sumit Rajaura, Harish Chandra, Ashutosh Singh, Ram Babu, Neelu Jain Gupta

**Affiliations:** a Department of Zoology and Environmental Science, Gurukula Kangri (Deemed to be University), Haridwar, Uttarakhand, India; b Department of Botany and Microbiology, Gurukula Kangri (Deemed to be University), Haridwar, Uttarakhand, India; c Department of Biochemistry, Lucknow University, Lucknow, Uttar Pradesh, India; d Department of Botany, Kirori Mal College, New Delhi, India; e Department of Zoology, CCS University Campus Meerut, Uttar Pradesh, India

**Keywords:** Benzo (a) pyrene, Epithelial cells, Inflammation, Lung cancer, Macrophages

## Abstract

This study was designed to investigate the toxic effects of benzo (a) pyrene (BaP) in the lungs. Mice were repeatedly treated orally with BaP (50 mg/kg body weight, twice a week for four weeks) to induce a tumour. After 4 months of BaP administration, tumours were visible beneath the skin. The histopathological section of the lungs shows congestion of pulmonary blood vessels, alveolar hyperplasia, and concurrent epithelial hyperplasia with infiltrates of inflammatory cells also seen. Thereafter, a single-cell suspension of lung tissues was stained with fluorescently conjugated antibodies for the demarcation of alveolar epithelial (anti-mouse CD74 and podoplanin) and macrophage (F4/80 and CD11b) cells and measured by flow cytometry. The expression of antioxidant genes was assessed by qRT–PCR. The number of alveolar epithelial cells 1 (AEC1) increased, but the number of alveolar epithelial cells 2 (AEC2) and transitional alveolar epithelial cells (TAEC) was significantly decreased in tumour-bearing mice. The proportion of CD11b^+^ alveolar macrophages (AM) and interstitial macrophages (IM) was increased, but the proportion of F4/80^+^ AM cells was reduced. The BaP administration significantly increased the ROS production in alveolar cells. The relative expression levels of antioxidant genes (SOD1, catalase, GPX1, and HIF-1α) were increased, but NRF2 expression was decreased in BaP-treated alveolar cells. The expression of anti-inflammatory (NF-κB) was also significantly increased. In conclusion, BaP exposure induced an inflammatory response, altered alveolar epithelial cell and macrophage diversity, and increased antioxidant responses in the lungs.

## Highlights

• Benzo (a) pyrene (BaP) produced a marked inflammatory response in mouse lungs.
• BaP treatment alters the diversity of macrophages and alveolar epithelial cells.
• BaP administration induces the ROS production in alveolar cells.
• BaP treatment increases the antioxidant and anti-inflammatory gene expressions.

## Graphical Abstract

**Figure:**
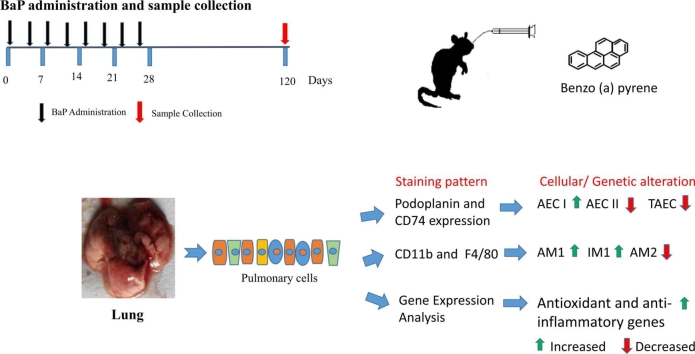

## 1 Introduction

The most common malignant tumour with a high fatality rate is lung cancer. The main causes of lung cancer are tobacco inhalation, chronic inflammation, and oxidative stress. It is becoming more common globally, with an annual increase of 0.5 %. In developed countries, men are more susceptible to lung cancer, mostly because of smoking [1]. Polycyclic aromatic hydrocarbons (PAHs), which are among the more than 60 carcinogens in tobacco smoke, are critical in the development of lung cancer [2], [3], [4]. Among PAHs, benzo (a) pyrene (BaP) is one of the most potent carcinogens that cause the development of lung cancer [5]. BaP is metabolically activated into an epoxide derivative, which reacts with DNA and is combined to form a DNA adduct that induces carcinogenesis [6], [7], [8]. It leads to oxidative damage to DNA by inducing reactive oxygen species (ROS) [9].

The initiation of a tumour stimulates immune responses within the lungs. The defensive cellular milieu in the lungs includes neutrophils, dendritic cells, alveolar epithelial cells (AECs), and alveolar macrophages (AMs). The activated neutrophil secretes a variety of pro-inflammatory mediators, which help in the killing and removal of pathogens [10]. The AMs survive longer and replenish 40 % in a year [11]. AMs represent the first line of defence and are phagocytic and antigen-presenting cells that remove cellular debris and apoptotic cells and elicit immune responses. They exhibit structural and functional plasticity in various diseases and function as immune regulators by secreting cytokines [12]. Macrophages can be classified into two main categories: M1 macrophages that are typically activated by IFN-γ and M2 cells that are alternatively triggered by IL-4. In contrast to M2 macrophages, which are both pro- and anti-inflammatory, M1 macrophages promote inflammation [13]. AMs are closely associated with AECs and dendritic cells and communicate with the AECs to initiate immunosuppression in inflammatory conditions [14]. The alveolar epithelium serves two important functions: it acts as a barrier and produces pulmonary surfactant. The AECs also secrete a range of mediators in response to pro-inflammatory agent stimulation [15]. The AECs are numerous, line the pulmonary airways and alveoli, and serve as a physical barrier to protect against respiratory infections. Two types of AECs are present: AEC1 and AEC2. About 95 % of the alveolar surface area is covered by the AEC1, which are terminally differentiated squamous epithelial cells [16]. The AEC2 covers about 4 % of the alveolar surface and helps in the maintenance of alveolar surface tension and immune regulation [17].

Multiple studies have delved into unraveling the immunosuppressive effects of BaP [18], [19]. Despite the well-known cellular composition of the lungs, a mouse model of cancer shows a distinct immune cell composition in the tumour microenvironment [20]. The role of AECs and AM_S_ in tumorigenic conditions has not been elucidated yet. The current study aims to elucidate the diversity of AECs and AMS in tumorigenic conditions by examining the cellular and molecular changes in pulmonary cells induced by BaP. This will be achieved through a detailed analysis of the toxic effects of BaP using histopathological, flow cytometric, and gene expression techniques.

## 2 Materials and methods

### 2.1 Animals

The present study was performed on Swiss male mice (6 in control and 7 in Bap-treated group) (10–12 weeks old, 30–35 g body weight). All research was done in accordance with the standards established by the Committee for the Purpose of Control and Supervision of Experiments on Animals (CPCSEA) and complied with the Animal Research: Reporting of *In Vivo* Experiments (ARRIVE) guidelines. The research was properly approved by the GKV Animal Ethics Committee (IAEC Code: GKV/AHF/14/2020). The animals were housed in microbe-free, positive-pressure air-conditioned facilities at GKV with a 12-h light/dark cycle, temperatures of 25 °C, and 60 ± 10 % humidity. The animals were continuously provided with clean drinking water and mouse food. The animals were sacrificed *via* cervical dislocation. The experiment was planned to use the fewest possible mice, and every attempt was made to minimize pain.

### 2.2 Chemicals and antibodies

PE anti-mouse Podoplanin and Alexa Fluor 647 anti-mouse CD74 (CLIP), FITC anti-mouse CD11b, APC anti-mouse F4/80, APC rat IgG2bκ, FITC rat IgG2bκ, PE rat IgG2bκ, were purchased from Bio Legends (San Diego, CA, USA). Purified rat anti-mouse CD16/CD32 was obtained from BD Biosciences (San Diego, CA, USA). ROS measurement dye 5 (and 6) chloromethyl-2′, 7′ dichlorodihydrofluorescein diacetate (CM-H_2_DCFDA) (D6883) was obtained from a molecular probe, Invitrogen (Eugene, OR, USA). Primescript™ First Strand cDNA Synthesis Kit, TB Green Premix Ex Taq, and PCR Master Mix were from Takara (Kyoto, Japan). Sigma-Aldrich (India) provided the benzo (a) pyrene, RPMI, HEPES, and TRI reagents. Fetal bovine serum (FBS) was purchased from Himedia (South Logan, UT). All primers were commercially synthesised by Eurofins. Primer sequences have been shown in Table 1.

**Table 1:** Sequences of primers used in the experiment.

S.No.	Gene	Forward primer (5’→ 3’)	Reverse primer (5’→ 3’)
1.	SOD1	CCATCAGTATGGGGACAATACA	GGTCTCCAACATGCCTCTCT
2.	SOD2	GACCCATTGCAAGGAACAA	GTAGTAAGCGTGCTCCCACAC
3.	CAT	CTCAGGTGCGGACATTCTATAC	GACTCCATCCAGCGATGATTAC
4.	Gpx1	GGAGAATGGCAAGAATGAAGA	CCGCAGGAAGGTAAAGAG
5.	HIF-1α	GGTTCCAGCAGACCCAGTTA	AGGCTCCTTGGATGAGCTTT
6.	NRF2	CACATCCAGTCAGAAACCAGTGG	GGAATGTCTGCGCCAAAAGCTG
7.	NF-κB	GAAATTCCTGATCCAGACAAAAAC	ATCACTTCAATGGCCTCTGTGTAG
8.	18s rRNA	ACTTTTGGGGCCTTCGTGTC	GCCCAGAGACTCATTTCTTCTTG

The sequences of primers used are shown.

### 2.3 Development of mice model of cancer by BaP administration

**Fig. 1:**
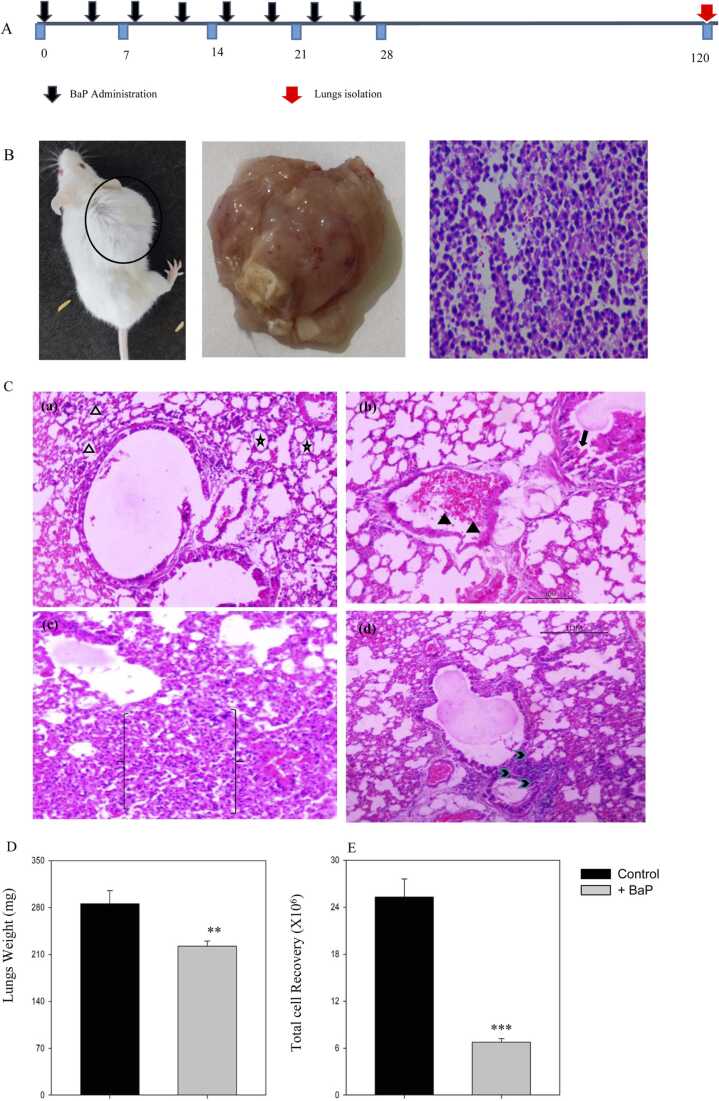
**BaP-induced tumour altered the lung’s histopathological architecture and reduced pulmonary cell recovery.** Mice were treated with BaP (50 mg/kg of body weight) twice a week for 4 weeks. (A) The detailed experimental protocol is shown in the line diagram. (B) Representative tumour bearing mice and histopathology section of tumour is shown. (C) The sections were prepared using a microtome and stained with haematoxylin and eosin. Light micrographs of the lung sections from the control have been shown in panel a. Panels b to d show the histopathological alterations observed in BaP-treated groups. (D) Shown here are the changes in total lung weight in control and treated groups. (E) Single-cell suspensions of lungs were prepared, and total cells were counted with the help of haemocytometer. Total cell recovery has been shown here. (a) The blank triangle shows normal alveolar wall thickness; star indicates normal alveolar architecture; (b) black triangle represents congested pulmonary blood vessels, black arrow shows irregular papillae lined by hyperplastic epithelial cells; (c) the area in between brackets shows alveolar hyperplasia with infiltrates of inflammatory cells; (d) the black arrowhead represents epithelial hyperplasia with marked inflammatory cells aggregate around it. Data is represented as mean ± SEM. n = 6 in control and 7 in treated groups. **p < 0.005, ***p < 0.005, student’s *t*-test, magnification 10 ×, Scale bar 1 µm.

Mice were treated with BaP dissolved in corn oil (50 mg/kg of body weight, twice a week for 4 weeks) *via* oral gavage; the control group received a vehicle alone [21], [22], [23] (Fig. 1). After 4 months, mice were dissected, lungs were excised, and single-cell suspensions were suspended in RPMI media containing 10 % FBS. Total cell recovery was enumerated using the Neuberger haemocytometer.

### 2.4 Histopathological analysis

Lungs from the control and BaP-treated mice were embedded in paraffin after fixation in 10 % formalin. The tissue 5 µm) were isolated from the embedded paraffin block using a rotary microtome. The tissues were deparaffinised and stained with haematoxylin and eosin. The slides containing sections were dehydrated, cleared, and mounted. Alterations in sections were then studied using a light microscope. The lungs were histopathologically examined [24], [25].

### 2.5 Flow cytometry analysis

None.

### 2.6 Analysis of ROS production

ROS level in alveolar cells was analysed by suspending the cells with 5-(and 6-)-chloromethyl-2’7’-dichlorodihydrofluorescein diacetate (CM-H_2_DCFDA). The oxidative conversion of CM-H_2_DCFDA into its fluorescent product was quantified by flow cytometry [26], [27], [28].

### 2.7 Analysis of alveolar epithelial cells

Alveolar epithelial cells (AECs) were recognised based on the expression of CD74 and podoplanin surface markers. A single cell suspension from the lungs was incubated with anti-CD16/32 antibody (Fc block, 1 µg/10^6^ cells in 50 µL of PBS + 2 % FBS) for 10 min, followed by labelling with anti-mouse podoplanin and anti-mouse CD74 antibodies, and then analysed on a flow cytometer [29], [30], [31].

### 2.8 Demarcation of alveolar macrophages

For enumerating the different types of alveolar macrophages (AM), single-cell suspensions were incubated with anti-CD16/32 antibody (Fc block, 1 µg/10^6^ cells in 50 µL of PBS + 2 % FBS) for 10 min. After staining with anti-mouse CD11bFITC and the F4/80 pan macrophage marker, cells were analysed on a flow cytometer [32], [33].

A BD FACS Verse flow cytometer was used for all flow cytometric studies, and Facsuite software was used for analysis.

## 3 Reverse transcription and quantitative real-time PCR

### 3.1 RNA isolation and cDNA synthesis

Total RNA was extracted from 1 × 10^6^ pulmonary cells by using Trizol reagent (Sigma). The RNA pellet was twice cleaned with 75 % ethanol, allowed to air dry, and then suspended in nuclease-free water. By analysing the absorbance at 260/280 and 260/230 nm wavelengths in a nanodrop spectrophotometer, the purity of the extracted RNA was determined. The RNA integrity was examined by running 5 µg of RNA on a 1.2 % formaldehyde agarose gel. 1 µg of RNA was used to make cDNA for RT-PCR [34], [35], [36].

### 3.2 Gene expression analysis using qPCR

The cDNA amplification was done by RT-qPCR. The expression levels of the antioxidant genes superoxide dismutase 1 (SOD1), superoxide dismutase 2 (SOD2), catalase (CAT), glutathione peroxidase 1 (GPX1), hypoxia-inducible factor 1α (HIF-1α), Nuclear factor erythroid 2-related factor 2 (NRF2), and anti-inflammatory nuclear factor kappa B (NF-κB) were analysed. mRNA levels were quantified by quantitative PCR (RT-qPCR) using Applied Biosystems QuantStudio 3, using SYBR green methods as per the manufacturer’s protocols [37], [38], [39], [40]. Samples were analysed in triplicate and normalized to 18S rRNA expression using the 2^−ΔΔCt^ method [41].

### 3.3 Statistical analysis

Sigma Plot 10 software was used to statistically analyse the data. The mean ± standard error of the mean (SEM) is used to express all data. For each variable that was measured, a Student *T*-test was used to compare the significant difference between the BaP-treated and the control group (untreated). A p-value less than 0.05 was considered significant.

## 4 Results

BaP treatment induced inflammation inside the lungs. The repeated BaP administration leads to the development of tumour beneath the skin (Fig. 1B). The morphological and histopathological alterations in the lungs were studied in BaP-induced tumour-bearing mice. A normal lung architecture without alterations in bronchioles, blood vessels, alveolar sacs, alveoli, or pneumocytes was seen in control mice (Fig. 1C, panel a). BaP administration causes congestion of pulmonary blood vessels with hyperplastic epithelial cells (Fig. 1C, panel b). The alveolar hyperplasia with infiltrates of inflammatory cells is also seen (Fig. 1C, panel c). The histopathological section also shows acute concurrent epithelial hyperplasia as recognised by the thickening of smooth muscles around the bronchiole in BaP-treated lungs (Fig. 1C, panel d). Further, we have also analysed the changes in weight and total cell recovery from the lungs of BaP-treated mice. The results show that the mean lung weight was 285 mg in control mice, which decreased to 218 mg after BaP treatment. A 24 % reduction in lung weight was observed (Fig. 1D). The results show that the mean cell recovery from the lungs also decreased from 245.25 million to 66.83 million (Fig. 1E).

### 4.1 BaP-induced tumours altered the alveolar epithelial cell diversity

**Fig. 2:**
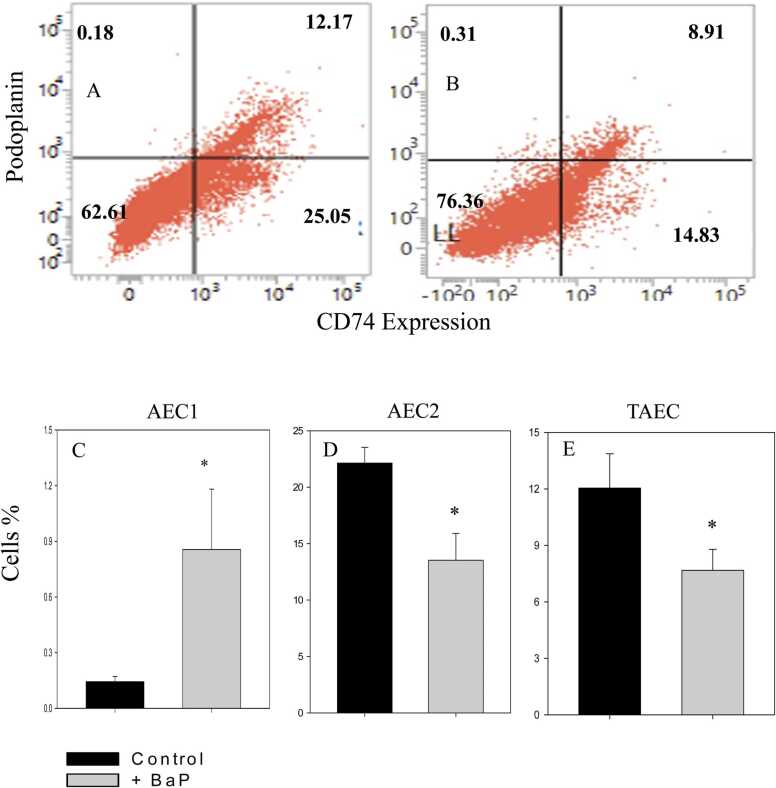
**Alteration in alveolar epithelial cells.** Mice were treated with BaP as described in the legend of Fig. 1. After induction of tumour, lungs were excised, and single-cell suspensions were prepared. Cells were stained with anti-mouse podoplanin and anti-mouse CD74 antibodies, followed by flow cytometric analysis. The flow cytometric dot-blots in panels A and B show the proportion of different types of epithelial cells in control and BaP-treated mice. The bar graphs in panels C–E show the cumulative proportion of AEC1, AEC2, and TAEC. Data is represented as mean ± SEM. n = 6 in control and 7 in treated group. *p < 0.05, student’s *t*-test.

Three different populations of alveolar epithelial cells can be recognised based on podoplanin and CD74 expression. These include AEC1 (podoplanin^+^), AEC2 (CD74^+^), and TAEC (podoplanin^+^CD74^+^). A representative flow cytometry histogram showing different types of epithelial cells is shown in Fig. 2. The data suggest that BaP administration significantly altered the proportion of various types of epithelial cells inside the lung. As depicted by the histogram, AEC1 increased from 0.18 % to 0.31 % after BaP administration. The proportion of AEC2 decreased from 25.05 % to 14.83 %, while the proportion of TAEC decreased from 12.17 % to 8.91 % (Fig. 2A and B). The cumulative data show a 7.5-fold increment in AEC1, a 39 % decline in AEC2, and a 34 % reduction in TAEC in the tumour-bearing mice (Fig. 2, panels C–E).

### 4.2 BaP-induced tumours altered the alveolar macrophage diversity

**Fig. 3:**
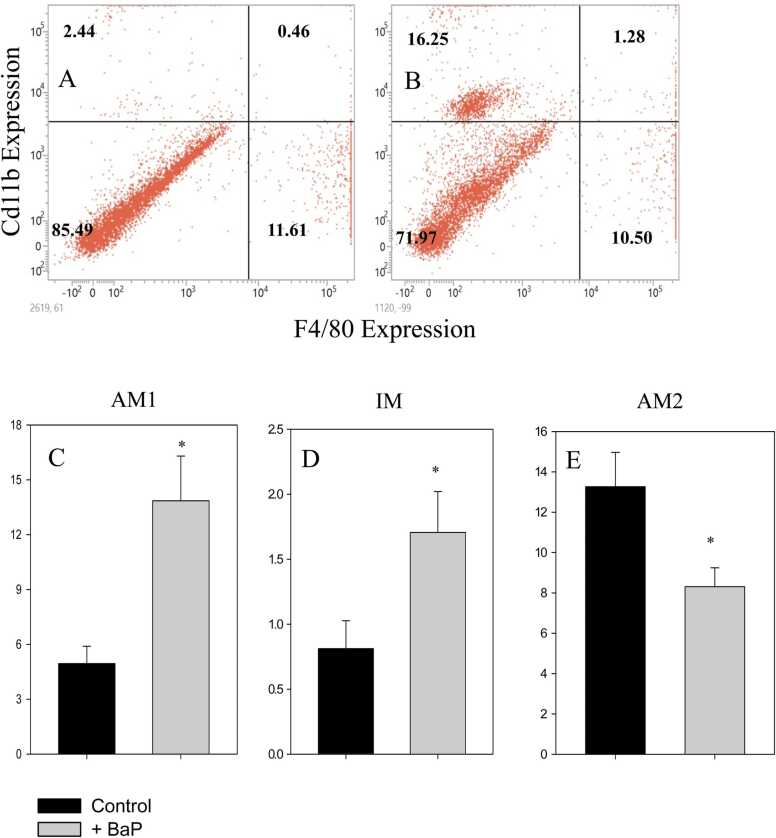
**Alteration in alveolar macrophages.** A single-cell suspension from BaP-treated lungs was stained with anti-mouse CD11bFITC and F4/80 APC, followed by flow cytometric analysis. The flow cytometric dot-blots in panels A and B show the proportion of different types of macrophages in control and BaP-treated mice. The bar graphs in panels C, D, and E show the cumulative changes in the proportion of AM1, IM and AM2 macrophages. Data is represented as mean ± SEM; n = 6 in control and 7 in treated groups. *p < 0.05, student’s *t*-test.

The impacts of BaP on macrophage cells in the lungs were studied by staining the cells with the CD11b/F4/80 pan macrophage markers [30]. Three types of macrophage cells, *i.e.,* alveolar macrophages 1 (CD11b^+^, AM1), interstitial macrophages (IM) (CD11b^+^F4/80^+^, IM), and alveolar macrophage 2 (F4/80^+^, AM2), were recognised (Fig. 3A and B). The representative histogram suggests that CD11b^+^ AMs and CD11b^+^F4/80^+^, IM were 2.44 % and 0.46 %, respectively in control mice, which increased to 16.25 % and 1.28 % in BaP-induced tumours. The proportion of AM2 was reduced from 11.61 % to 10.50 % after tumour induction. Overall, the cumulative data suggests AM1 and IM show 2.8- and 2.1-fold increments after induction of tumour, respectively (Fig. 3, panels C and D). The AM2 cells were 38 % reduced in the BaP-induced tumour mice model (Fig. 3E).

### 4.3 BAP-induced ROS production in lungs

**Fig. 4:**
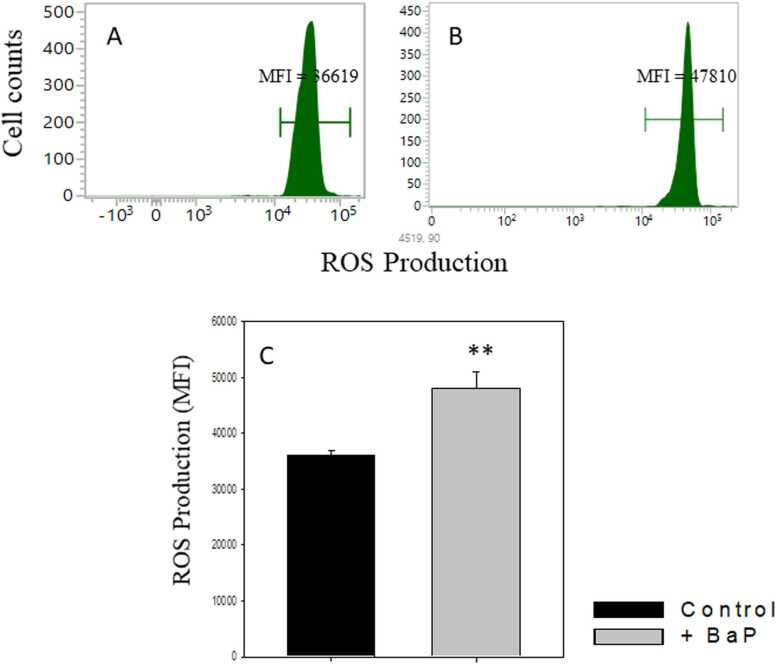
**BPA administration increased the ROS** expression in alveolar cells. Mice were treated with BaP as shown in the legend of Fig. 1. After induction of tumour, lungs were excised, and single-cell suspensions were prepared. The ROS production in alveolar cells was analysed by CMH_2_DCFDA staining followed by flow cytometry. Panels A, and B represent ROS MFI level in control and BPA treated alveolar cells, respectively. The cumulative changes in ROS MFI level have been shown in panels C. Data is shown as mean ± SEM. n = 6 in control and 7 in treated group. **p < 0.005, student’s *t*-test.

The changes in ROS production in BPA-treated alveolar cells are depicted in Fig. 4. BPA treatment induces the ROS level in alveolar cells. The representative histograms suggest that mean fluorescence intensity (MFI) of ROS production was 36,619 which is increased to 47,810 after BaP administration (Fig. 4 Panels A and B). The cumulative data show that ROS MFI was significantly increased (33 % more than control) in BaP-treated alveolar cells (Fig. 4C).

### 4.4 Changes in the relative expression of antioxidant genes

**Fig. 5:**
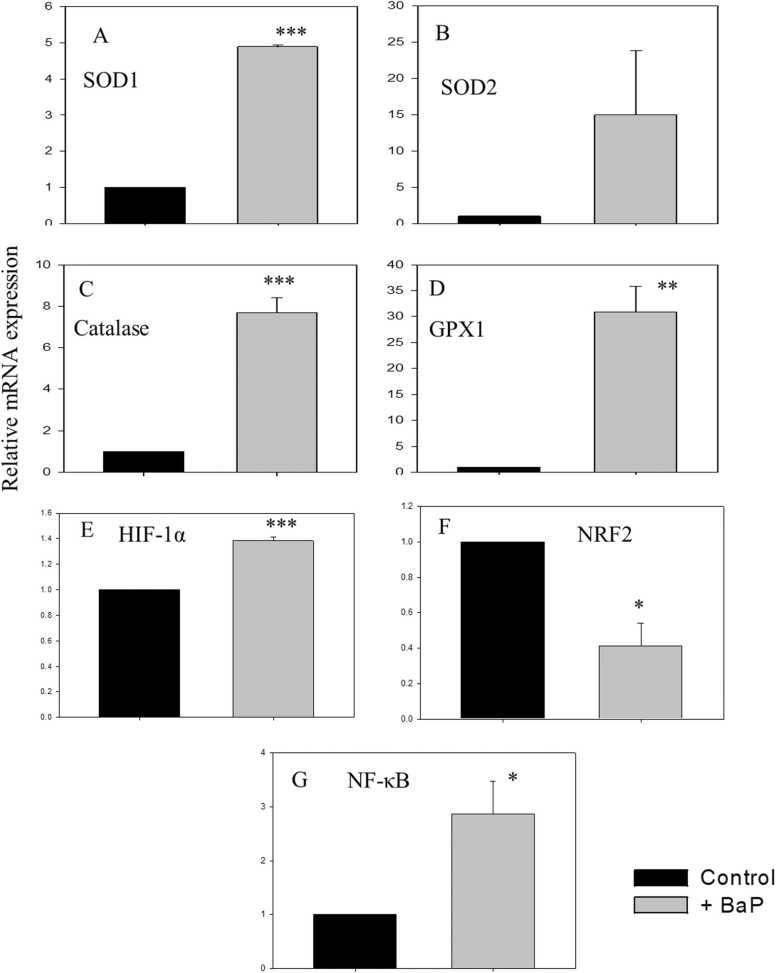
**The BaP administration increased the expression of antioxidant genes expression in alveolar cells.** RNA was isolated from pulmonary cells by using Trizol (TRI) reagents. The first-strand cDNA was prepared by utilizing the First Strand cDNA Synthesis Kit and amplified by qRT-PCR. The RNA transcripts for different genes were measured by the SYBR Green method. Relative expression levels of antioxidant genes SOD1, SOD2, CAT, and GPX1 have been shown in panels A to D. Panels E to G depicts the changes in HIF-1α, NRF2and NF-κB expression. The mRNA levels were normalized to 18S rRNA levels using the 2-^ΔΔC^T methods. Data is represented as mean ± SEM of three independent experiments. *p < 0.05**p < 0.005, ***p < 0.0005, student’s *t*-test.

The relative expression of antioxidant genes (SOD1, SOD2, CAT, GPX1, HIF-1α, and NRF2) and anti-inflammatory (NF-κB) was analysed in BaP-treated lungs. The data suggests that the expression of antioxidant genes was significantly increased. The relative expression levels of SOD1 (4.89 ± 0.04), CAT (7.69 ± 0.70), GPX1 (30.90 ± 4.95), and HIF-1α (1.38 ± 0.03) mRNA were significantly increased, but NRF2 expression was decreased (Fig. 5, panels A–F). BaP administration also significantly increased the NF-κB (2.86 ± 0.61) expression in alveolar cells (Fig. 5G).

## 5 Discussion

Lung cancer accounts for 20 % of all cancers and has a global death rate of 18.4 %. Patients with lung cancer had a 56 % 5-year survival rate, which falls to 5 % in malignant instances [42]. Generally, addicted smokers and ex-smokers are at great risk of contracting lung cancer since 90 % of lung carcinogenesis is caused by tobacco smoke exposure [43]. Among more than 60 known carcinogens in tobacco smoke, PAHs are particularly important in the development of lung cancer [44]. In the current investigation, we looked at immunosuppression in lung cancer caused by the carcinogen BaP, which is considered a marker of the carcinogenic potency of PAHs by the WHO [45]. BaP-derived epoxide reacts with DNA to form an adduct that allows carcinogenesis to progress [46].

Firstly, we studied the alterations in lung morphology and histopathology after the development of the tumour. The lungs in tumour-bearing mice were enlarged, turgescent, and appeared dusky red in colour. The lung’s histopathological observations represent normal bronchioles, blood vessels, alveolar sacs, alveoli, and pneumocytes in control mice. The BaP administration causes cell injury and inflammation, which leads to concurrent epithelial hyperplasia. The inflammatory mononuclear cell infiltration usually occurs in response to toxicants, necrosis, and pulmonary neoplasms [47]. The histopathological section also shows irregular papillae and congestion in pulmonary vessels, which indicates an injury to the bronchi in BaP-treated mice [48]. The alveolar hyperplasia suggests abnormal proliferation of the terminal bronchiolar epithelium.

The decline in lung weight is due to a reduction in cell number and inflammation. Inflammatory responses and edema can also lead to enlarged lungs, and these conditions may involve increased organ size without a proportional increase in cell size and weight. A significant decrease in pulmonary cell recovery was observed in tumorigenic mice. Overall, histological and cytological studies show a series of alterations that occur over time and represent a morphological progression to bronchogenic cancer.

A well-characterised cellular system with more than 40 types of cells, including neutrophils, dendritic cells, AECs, and AMs, is present inside the lungs [49]. We estimated the heterogeneity of AECs by analysing the surface expression of podoplanin and the CD74 receptor. Our data show a marked change in the proportion of various AECs in tumorigenic lungs. AEC1 was significantly increased, while AEC2 and TAEC were significantly reduced. AEC2 has been shown to regulate surfactant metabolism, ion transport, immune defence, and lung injury repair within the lungs [50]. The AEC2 have the ability to self-renew and also serve as progenitors for the AEC1 cells. Since our histopathological observations suggest that BaP administration causes inflammation and lung injury, the AEC2 helps in the repair mechanism and declines accordingly. The TAEC represents intermediate cells between AEC1 and AEC2. The exact mechanism of TAEC is not clear; however, it may be possible that the proportion of these cells declined as these cells were transformed into AEC1 cells. Overall, the heterogeneity of AEC was altered in BaP-treated lungs.

The destruction of AECs triggers the macrophages to initiate an immune response [51]. We have studied the macrophage response based on CD11b/F4/80 staining. Based on the surface expression analysis, three different types of macrophages were recognised, *i.e.,* AM1, CD11b^+^, IM CD11b^+^F4/80^+^, and AM2, F4/80^+^. In tumorigenic lungs, the proportions of AM1 and IM were significantly increased, while AM2 cells were significantly decreased. CD11b, also known as integrin αM, is a component of the heterodimer integrin M2 (macrophage-1 antigen) and is one of the most effective molecular markers of the macrophage lineage [52]. CD11b is highly expressed in tumour-associated macrophages and is linked to chronic and acute lung inflammation [32]. AM1 plays a crucial role in the immune response in the lungs. They are responsible for phagocytosis, antigen presentation, cytokine production, and continuously monitoring the lungs for pathogens and foreign substances. They play a complex role in tumor development and cancer progression in the lungs. The increased proportion of AM1 and IM that we have observed will suppress the adaptive immune response and promote tumour growth. The AMs release pro-inflammatory cytokines and inhibit tumour growth. Earlier research showed that AMs serve an important function in cancer metastasis and inhibit adaptive immune responses [53]. AM2 are involved in the resolution of inflammation and the prevention of excessive immune response. Our results show a 38 % reduction in AM2 cells, which is correlated with earlier observations. It is reported that the decline in AM2 led to the increment of CD4^+^ T cells and the maturation of DC cells inside the lungs, which initiated an adaptive immune response [54]. Overall, the immunophenotyping data suggests that the proportion of AMs and AECs changed in the tumour-bearing mice.

It is reported that the tumour microenvironment and inflammation cause increased ROS production, which may have a deleterious impact on the lungs [55]. We have also observed increased ROS production in BaP treated alveolar cells. BaP administration can increase ROS production *via* mitochondrial dysfunction by the impairment of the electron transport chain. This could be the possible reason for the induced inflammation in BaP-treated lungs. Earlier, it was also reported that BaP treatment causes mitochondrial dysfunction and induces the ROS production in human lung cancer cells [56].

A pool of antioxidant enzymes is present in alveolar cells, which maintains redox balance. They help in neutralizing ROS and protect cells from oxidative damage. We analysed the changes in antioxidant (SOD1, SOD2, CAT, GPX1, HIF-1α, and NRF2) and anti-inflammatory nuclear factor kappa B (NF-κB) gene expression in tumour-bearing mice. SOD1, CAT, GPX1, and HIF-1α were significantly increased in the lungs of tumour-bearing mice. The observed increase in antioxidant gene expression may be part of the host's defence against the inflammatory environment associated with the presence of tumours. It allows the cells to avoid apoptosis caused by the excessive production of ROS while maintaining the radicals that are required for cell proliferation and metastasis [57]. Further, the increase of these enzymes could lead to protection from oxidative damage and also help in the inhibition of tumour growth inside the lungs [58]. NRF2, a critical transcription factor regulating antioxidant defence mechanisms, is conversely downregulated in response to BaP treatment. Under normal conditions, NRF2 binds to antioxidant response elements (AREs) to promote the transcription of detoxifying and antioxidant enzymes, maintaining cellular homeostasis. However, BaP impairs NRF2 activity, leading to diminished antioxidant defenses and heightened susceptibility to oxidative damage [59]. Studies involving NRF2-deficient models have further demonstrated increased vulnerability to BaP-induced carcinogenesis, underscoring the protective role of NRF2 in detoxifying harmful byproducts of BaP metabolism [60].

Additionally, NF-κB, a pivotal mediator of the inflammatory response, is also upregulated upon BaP exposure. BaP-induced ROS leads to the inhibition of IκB, an inhibitor of NF-κB, allowing the translocation of NF-κB to the nucleus, where it activates the transcription of pro-inflammatory and immune-related genes. The simultaneous upregulation of antioxidant genes and NF-κB suggests that BaP exposure induces an inflammatory response coupled with survival pathways, potentially contributing to carcinogenesis and tissue damage, as observed in various BaP-exposure models [61], [62]. Altogether, these findings highlight the intricate play between lung tumours and the host's molecular defence mechanisms in the context of oxidative stress.

## 6 Conclusions

The study reveals that BaP administration leads to tumor development, causing epithelial hyperplasia, pulmonary blood vessel congestion, and inflammatory mononuclear cell infiltration. This microenvironment alters AMs and AECs diversity, leading to immunosuppression. The expression of antioxidant and anti-inflammatory genes was also significantly increased in the lungs of BaP-treated mice.

## CRediT authorship contribution statement

**Ashutosh Singh:** Writing – review & editing, Writing – original draft, Investigation, Formal analysis. **Harish Chandra:** Writing – review & editing, Writing – original draft, Investigation, Formal analysis. **Sumit Rajaura:** Writing – review & editing, Writing – original draft, Investigation, Formal analysis, Data curation. **Nitin Bhardwaj:** Writing – review & editing, Writing – original draft, Resources, Project administration, Methodology, Investigation, Funding acquisition, Formal analysis, Data curation. **Neelu Jain Gupta:** Writing – review & editing, Writing – original draft, Formal analysis, Data curation. **Pooja Chauhan:** Writing – review & editing, Writing – original draft, Investigation, Formal analysis, Data curation. **Ram Babu:** Writing – review & editing, Writing – original draft, Data curation.

## Author Contributions Statement

All authors contributed to the study conception and design. Material preparation, data collection and analysis were performed by Pooja Chauhan, Nitin Bhardwaj and Sumit Rajaura, Ashutosh Singh, Rambabu, Neelu Jain Gupta and Harishchandra. The first draft of the manuscript was written by Pooja Chauhan, Nitin Bhardwaj and Sumit Rajaura and all authors commented on previous versions of the manuscript. All authors read and approved the final manuscript.”

## Ethical Approval

The research was properly approved by the GKV Institutional Animal Ethics Committee (IAEC Code: GKV/AHF/14/2020).

## Conflict of interest statement

The authors declare that they have no competing interests.

## Declaration of Competing Interest

The authors declare the following financial interests/personal relationships which may be considered as potential competing interests: Nitin Bhardwaj reports financial support was provided by Indian Council of Medical Research. Nitin Bhardwaj reports financial support was provided by University Grant commission. If there are other authors, they declare that they have no known competing financial interests or personal relationships that could have appeared to influence the work reported in this paper.

## Data Availability

Data will be made available on request.
